# Social factors contributing to the development of chronic low back pain: a scoping review

**DOI:** 10.1186/s12891-025-09216-4

**Published:** 2025-10-27

**Authors:** Yvan Sonjon, Annabelle Gonthier, Camille Lépingle, Jean-Baptiste Van der Henst, Karen T. Reilly, Eric Chabanat

**Affiliations:** 1 Centre Hospitalier Saint Jean de Dieu, Fondation ARHM, Fédération de Recherche, 69008 Lyon, France; 2 Centre de Recherche en Neurosciences de Lyon CRNL U1028, UMR5292, Université Claude Bernard Lyon 1, CNRS, INSERM, TRAJECTOIRES, 69500 Bron, France; 3 Institut Des Sciences Et Techniques de Réadaptation, Université Claude Bernard Lyon 1, 69008 Lyon, France; 4 Institut d’Histoire Des Représentations Et Des Idées Dans Les Modernités IHRIM UMR5317, Ecole Normale Supérieure de Lyon ENSL, CNRS, Lyon, France

**Keywords:** Chronic Low Back Pain, Social Factors, Bio-psycho-social model, Scoping review, Social support

## Abstract

**Background:**

Research and clinical practice approaches to chronic low back pain (CLBP) have predominantly focused on biomedical and psychological aspects, often overlooking social influences. The biopsychosocial (BPS) model provides a theoretical framework for conceptualizing CLBP as the outcome of complex interactions between individuals and their environment, offering valuable insights into its progression and informing effective care strategies. This scoping review seeks to identify social factors contributing to CLBP, and to propose a classification system that offers a complementary perspective on social influences.

**Methods:**

Eight databases were searched following the JBI methodological guidelines for scoping reviews. The reporting of this review is guided by the PRISMA-ScR guidelines. Sources were selected based on the Population-Concept-Context (PCC) framework, specifically targeting studies involving adults with CLBP assessed through the BPS model. The focus was on social factors linked to CLBP evolution in the context of Westernized countries. Two researchers independently screened articles for inclusion, extracted data, and conducted the analysis. The protocol for this review was prospectively registered (DOI: https://doi.org/10.17605/OSF.IO/8DBJV).

**Results:**

Among the 35 articles meeting inclusion criteria, we identified 28 categories of social factors potentially associated with CLBP. These factors were organized into six domains spanning from individual-level influences to broader societal contexts.

**Conclusions:**

This review provides a comprehensive overview of social factors influencing CLBP. The proposed classification provides a foundation for future research and may assist clinicians in better understanding the social dimensions of chronic pain, ultimately contributing to the development of more personalised interventions tailored to patients' social and life contexts.

**Supplementary Information:**

The online version contains supplementary material available at 10.1186/s12891-025-09216-4.

## Background

In 1977, psychiatrist George Engel introduced the biopsychosocial (BPS) model, stating that understanding illness "must take into account the patient, the social context in which he lives, and the complementary system devised by society to deal with the disruptive effects of illness." [1]. Initially introduced in psychiatry, the model was later extended to the study of pain, in particular low back pain, by John Loeser and Gordon Waddell, who, upon observing the ineffectiveness of surgical treatments for this condition, highlighted its value in explaining chronic low back pain (CLBP) [2, 3], which cannot be reduced to nociceptive activity [4] or mechanisms such as central sensitisation [5, 6]. Despite its promise, the BPS model has been slow to be adopted, likely because the deeply entrenched biomedical model in Western healthcare systems imposes institutional constraints and continues to shape funding and health policy decisions, thereby limiting the broader adoption of the BPS model, which is essential for managing chronic conditions [7].

Current guidelines acknowledge the importance of the biological, psychological, and social dimensions by recommending a BPS framework for CLBP management [8]. However, these dimensions are mostly kept separated [9], with a hierarchy that minimises the importance of social factors [10]. Furthermore, the development of the BPS model has been accompanied in recent years by increased attention to the psychological dimensions of pain, both in research and clinical practice [11, 12]. Despite these recent advances, the focus tends to remain primarily on internal factors specific to the individual, such as cognitive and emotional factors (e.g. beliefs and thoughts), like catastrophizing, fear-avoidance and fear of movement [13–15]. In contrast, social factors that represent an external dimension to the individual have received comparatively less attention and are still under-explored in the context of CLBP [16]. These factors can be understood at two levels: local and direct and structural and indirect. The first level refers to direct influences from the individual's immediate environment, such as family, friends, or colleagues. They are relatively malleable and can often be modified through individual actions or even clinical interventions [17–19]. Structural influences, by contrast, operate at a societal level and refer to broader contexts such as social class, cultural standards related to occupational activities, as well as institutional and power structures that establish norms and behavioural expectations [17, 20, 21]. These influences shape individual behaviours by establishing the social and economic conditions in which people live. Addressing them often requires interventions at the community level or public health policies, since individual agency is more limited. As chronic pain reflects population health and well-being, integrating social perspectives from individual to societal levels could improve health assessments and deepen understanding of how social structural dynamics contribute to CLBP [22].

Adverse social experiences, such as rejection or social downgrading, can contribute to dysregulation of the immune and endocrine system functions or impair emotional processing, thereby increasing chronic pain risk [23]. Moreover, the relationship between chronic pain and social factors, may be affected by social threats that undermine basic human needs [24]. When individuals with chronic pain no longer conform to the social norms of their environment, they may be perceived as less warm and competent, increasing their risk of isolation or exclusion [25]. Such negative perceptions can trigger stigma and discrimination, intensifying emotional distress and perceived pain, and contributing to a cycle of marginalisation described as a social threat [24].

Compared to the other two dimensions of the BPS model, the literature addressing social factors in chronic pain appears less developed and limited in scope. Aspects such as interpersonal and professional relationships, socioeconomic precarity, or working conditions are already recognized as influential in shaping the experience of chronic pain, particularly in relation to progression to a chronic state, quality of life, or return to work [26–28]. When considered, these factors are often treated as individual characteristics, without an explicit modelling of their structural, relational, or institutional embeddedness [29]. Although one review adopts a systemic perspective on the social repercussions of chronic pain [30],such approaches remain uncommon in the existing literature. This highlights the need to move beyond an individual-level perspective to conceptualize social factors as external and contextual contributors to chronic pain, and to expand their analysis toward more structural and systemic dimensions.

More specifically related to CLBP, a preliminary search in MEDLINE, the Cochrane Database, JBI Evidence Synthesis, and PROSPERO revealed one recent review on BPS prognostic factors in CLBP [31] and a pre-registered review on social determinants of health and acute low back pain onset [32]. However, the methodologies employed in the aforementioned reviews, due to restrictive inclusion criteria, may not have allowed for the exploration of an expanded selection of literature studying social factors, particularly from fields such as psychology, behavioural studies, or health sociology. To address these epistemological and methodological limitations, we opted for a scoping review, a methodology specifically designed to explore the types of evidence available in a given field [33]. The aim of this scoping review is to identify the wide range of social factors associated with CLBP, including those traditionally considered biological or psychological but understood within our conceptual framework as socially shaped. It also seeks to propose a classification system that offers a complementary perspective on social influences in the development of CLBP.

## Methods

This scoping review was conducted following the methodological guidance from the Joanna Briggs Institute (JBI) Manual for Evidence Synthesis: Scoping Reviews Guide and its update [34, 35]. The reporting of this review is guided by the PRISMA extension for scoping review guidelines and its associated checklist [36]. The protocol for this review was registered prospectively with the Open Science Framework on (January 18, 2024) (pre-registration 10.17605/OSF.IO/8DBJV).

### Eligibility criteria

*Participants*: Studies including adults with non-specific chronic low back pain (CLBP), defined by the National Institutes of Health as pain in the lumbar region present for at least 3 months (or 12 weeks), even after an initial injury or underlying cause of acute low back pain has been treated [37]. This includes cases where previous treatments, such as medication, rehabilitation, or surgery (e.g., Persistent Spinal Pain Syndrome Type 2 cases), have failed to provide relief. Studies were excluded if CLBP was attributed to specific underlying conditions, including systemic, inflammatory, tumoral, acute tissue injury (e.g., fractures), gastrointestinal, renal, infectious, or endocrine pathologies. Additionally, studies focusing on chronic cervical or thoracic pain were excluded. Only studies reporting CLBP as their primary focus were included, while studies combining CLBP with other chronic pain conditions were excluded unless separate data for CLBP was available. *Concept*: Studies investigating the transition to and evolution of CLBP through the lens of social factors, defined as external influences on the individual within the theoretical framework of GL Engel’s BPS model [1]. We considered factors to be social when they were shaped by direct interpersonal influences (e.g. family, social support, workplace relationships) or by structural influences (e.g. socio-economic status, healthcare accessibility, cultural norms). Within this framework, some factors frequently described as biological or psychological in the literature were regarded as social, as they are fundamentally shaped by social determinants and structural contexts. The inclusion criteria focused on research that explicitly integrated social factors into the assessment and understanding of CLBP, and factors that were explored for their predictive role in its evolution were highlighted. *Context*: Studies were included if they focused on research (e.g., clinical trials, literature reviews, qualitative studies) or the development of guidelines for the classification and management of CLBP. Additionally, studies examining healthcare practices in primary care, secondary care (e.g., rehabilitation in certain European countries), and tertiary care (e.g., specialized pain management centres) were also considered. Studies were included regardless of whether they were conducted in public or private healthcare settings. This scoping review focused on patient populations from industrialized Western countries, which share comparable relational, cultural, and political frameworks, as well as similar healthcare structures and delivery models.

### Types of Sources

If they met our inclusion criteria, defined by the Population, Concept, Context (PCC) framework, the following study designs were considered: experimental studies, including randomized controlled trials, non-randomized controlled trials, before and after studies and interrupted time-series studies. We also assessed analytical observational studies according to our PCC criteria, including prospective and retrospective cohort studies, case–control studies and analytical cross-sectional studies, descriptive observational study designs including case series, individual case reports and descriptive cross-sectional studies. Studies focusing on qualitative data derived from various approaches including (but not limited to) phenomenology, grounded theory, ethnography, qualitative description, action research and feminist research were examined and included when they met our PCC criteria. We also included systematic reviews, as well as text and opinion papers. Due to language constraints for interpreting results we only included studies published in English or French. To ensure an up-to-date vision of the state of knowledge in this field only studies published between 2010 and 2023 were included.

### Search strategy

An initial limited search of electronic databases MEDLINE and ScienceDirect was conducted by YS, AG and EC between October and November 2023 to identify articles on the topic. The text words contained in the titles and abstracts of relevant articles, and the index terms used to describe these articles were then used to develop a full search strategy for MEDLINE (see supplementary material, Additional file 1). The search strategy, including all identified keywords and index terms, was adapted for each search database and/or information source. A total of eight electronic databases were consulted by YS and AG between December 2023 and February 2024: Medline, Cochrane Library, Science Direct, Psychinfo, Cairn, Web of Science, JSTOR and Google Scholar. All database research equations can be found in the supplementary data (see Additional file 1). The reference lists of all included evidence sources were screened for possible inclusion of additional studies.

### Source of evidence selection

**Fig. 1:**
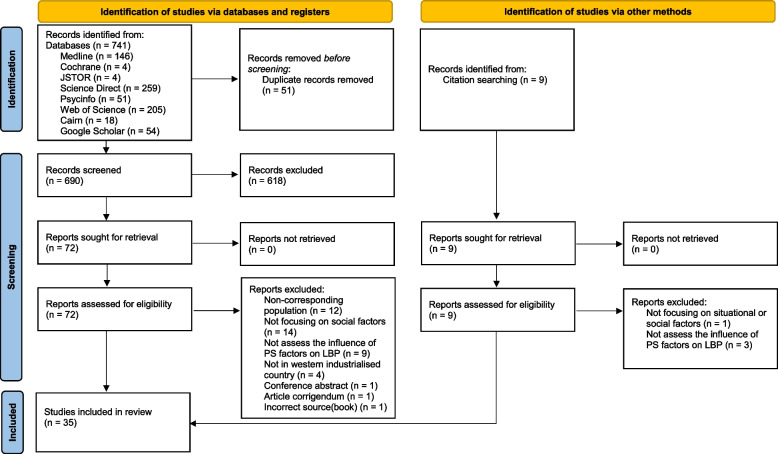
Flow diagram. This flow diagram follows the PRISMA 2020 template for new systematic reviews which included searches of databases, registers and other sources [74]

All identified articles were collated and uploaded into Zotero7 (2023), with duplicates removed. Following the recommendations of the Joanna Briggs Institute [34, 35] we performed an initial pilot test to ensure consistency in the screening process. Subsequently, all titles and abstracts were independently reviewed by two evaluators (YS and AG) with regards to the inclusion criteria. The full text of potentially relevant sources was then retrieved and citation details imported into our data-charting instrument (see Supplementary Material, Additional file 2). In a second screening step, the full text of each potentially relevant source was assessed in detail against the inclusion criteria by the same two reviewers who participated in step 1, each working independently. This led to the further exclusion of a number of citations (reasons for exclusion at this stage are reported below). Any disagreements that arose between the reviewers were resolved through discussion between the two initial reviewers in an effort to reach consensus. If consensus could not be achieved, an additional reviewer (EC) was consulted and a final decision was reached after discussion between the three reviewers. The results of the search and the study inclusion process are presented in Fig. 1 in the form of a Preferred Reporting Items for Systematic Reviews and Meta-analyses extension for scoping review (PRISMA-ScR) flow diagram [36].

### Data extraction

The data from the included articles were independently extracted by two reviewers (YS and AG), without prior consultation between them, using an in-house data extraction tool based on the JBI data extraction instrument template (latest version July 2022). To ensure the reliability of the process, the extraction method was tested on three randomly-chosen articles and the extracted data compared between reviewers. This initial step reveals only small discrepancies, and after discussion the data extraction method was agreed upon. After this, two reviewers (YS and AG) extracted the data from all included articles (see Supplementary Materials for the final data extraction form which lists all extracted data, Additional file 4, Additional file 5). As in the previous stage (Selection of Source of Evidence), any discrepancies between the two reviewers were systematically discussed in an attempt to reach a consensus. If consensus could not be achieved, a third reviewer (EC) was consulted and a final decision was reached after discussion between the three reviewers. The entire process of article inclusion and data extraction took place between January 2024 and May 2024.

### Data analysis and presentation

We first performed a descriptive statistical analysis of the included articles. Following this YS, AG and EC performed our main, basic qualitative content analysis by carefully examining the content of the articles using an inductive model [38]. This method was necessary in order to gain an overview and summarise the various factors identified using basic qualitative content analysis. Each author involved in this step independently analysed the extracted data, which were compiled in the data charting form (see Additional file 5). This document served as a support for data organization and classification.

In accordance with our conceptual framework (see Introduction), we defined social factors as external influences on the individual that arise from interpersonal, institutional, or societal contexts. These include both direct influences (e.g., family, colleagues, place of living) and indirect, structural influences (e.g., socioeconomic status, cultural norms, healthcare system organisation, work organisation) that shape health and behaviour through broader social conditions. This definition informed both our eligibility criteria and our inductive categorisation of social factors.

Each social factor identified during the data extraction process in the included articles was categorized by each author independently and without prior consultation with the other authors. This categorization was based on the factor’s description in the original article and each factor was grouped under a broader descriptor that encompassed similar factors. The categorisation process was conducted iteratively in regular meetings between the authors, during which they ensured that factors with equivalent meanings (based on examination of the description, the concept and the assessment behind each extracted factor) were grouped under a common broader descriptor term, a category. This classification was validated by consensus between the authors to ensure consistency in the organisation of the data and to facilitate the synthesis of the results. Category names were assigned by the authors who attempted to use names frequently used in the included studies, their ability to reduce ambiguity and limit misinterpretation, and their validation through author consensus.

During this initial phase of basic qualitative content analysis using an inductive approach, YS, AG, EC, and JBVDH held consensus meetings to compare individual analyses, identify discrepancies, and resolve them through discussion.

The resulting categorization system was then used to develop broader domains, in order to generate a structured synthesis of the categorized social factors. The naming of the domains was determined through consensus discussions among the authors (YS, AG, JBVDH, KR, and EC), ensuring their semantic relevance in representing the grouped categories. The domains were structured based on the relationships observed among the categories derived from the extracted data, in an attempt to ensure that the names reflected the overarching dimensions of social influences related to CLBP.

Given the diversity in the levels of evidence among the included studies (both quantitative and qualitative) and the exploratory nature of our basic qualitative content analysis, a critical appraisal of the studies was neither appropriate nor relevant to the aim of our review. This decision is consistent with the JBI recommendations for conducting scoping reviews [35, 39].

In order to check the completeness of our article selection method, YS and EC analysed the citation network of the included articles to determine whether our selection contained mainly articles that cited each other or whether we had managed to obtain a broader overview of the scientific literature. To do this we followed the open access procedure described on GitHub (https://github.com/jaks6/citation_map, see Supplementary Material, “Additional file 3”).

## Results

The search identified 741 articles. After removing duplicates and screening articles based on title and abstract, 72 articles were retained for full-text assessment. In addition, 9 articles were identified from the reference lists of other articles and were added in at the full-text review phase. Figure 1 shows the PRISMA flow diagram illustrating the article identification and inclusion process. A total of 46 articles were excluded after full-text reading: 12 with populations that did not correspond to our inclusion criteria, 15 that did not focus on social factors, 12 that did not assess the influence of psychosocial factors on CLBP, 4 reporting data in countries that did not meet our inclusion criteria, 1 conference abstract, 1 book chapter and 1 correction to an article that did not impact the analysis of our results. After the initial and full-text screening phases 35 articles met the inclusion criteria [31, 40–73]. Table 1 shows the characteristics of the included articles with respect to methodology, design, publication date, and geographical location.

**Table 1:** Characteristics of included articles

Study characteristics	n/35 (%)
**Methodology**	
Quantitative	24 (69)
Qualitative	8 (22)
Mixed or multi-methods	3 (9)
**Design**	
Prospective cohort	10 (28)
Cross sectional	4 (11)
Observational	3 (9)
Systematic review w/o Meta-analysis	7 (20)
Mixed method	3 (9)
Narrative review	4 (11)
Semi-structured qualitative interview	1 (3)
Qualitative evidence synthesis	3 (9)
**Publication Date**	
2010–2011	7 (20)
2012–2013	5 (14)
2014–2015	3 (9)
2016–2017	3 (9)
2018–2019	5 (14)
2020–2021	6 (17)
2022–2023	6 (17)
**Region/Country where the studies were conducted**	
USA	7 (20)
Europe	19 (54)
Australia	6 (17)
New Zealand	2 (6)
Canada	1 (3)

### Social factors

Table 2 contains a detailed summary of each of the 35 included articles (raw extracted data are provided in Supplementary material, Additional file 5). The column “ Social factors” contains the names of the various factors used by the authors of each study. Initial examination of this column reveals a large number of terms and concepts that upon closer examination often refer to similar factors. To simplify this terminological heterogeneity, which made it difficult to obtain a comprehensive overview of social factors, we grouped these terms into 28 distinct factor categories. For example, within the “Work Status’ category, the included studies reported a variety of terms such as: employed/not employed; work absence due to LBP/currently employed in normal job/currently employed on reduced duties because of LBP/currently unemployed because of LBP/currently unemployed because of other reasons. Table 3 describes the 28 different factor categories that emerged from analysis of the data in Table 2 and provides concrete examples from included studies. These factor categories were then further grouped into larger Social Domains, six of which emerged: socio-demographic (SD), socio-occupational (SO), socio-environmental (SE), global health data (GHD), psycho-social (PS), patient’s experience of health care (PEHC). While several of the included studies originally described these factors as biological or psychological, in line with our conceptual framework we categorised them as social factors. This reflects their contextual association with broader social determinants and systemic influences and is consistent with JBI methodological guidance and our basic qualitative content analysis (see Methods). It is important to keep in mind that this approach is descriptive in nature and does not reinterpret the findings of the original studies. More detailed descriptions of each Social Domain and the frequency of their representation within the included articles are provided below.

**Table 2:** Detailed summary of each of the included studies

Study	Methodology *Design*	Aim(s) of the study	Social factors (as named in the study)	Social factors assessment tools (w/o demographics)	Outcome for chronic low back pain evolution	Sample Size (inclusion status)
Aroke et al., 2020 [34]	Quantitative *Observational longitudinal study*	The objective of this study was to assess whether self-identified race moderated the relationship between perceived social status and cLBP outcomes (pain interference and pain severity) and investigate whether race moderated the indirect relationship between perceived social status and pain outcomes via depressive symptoms	Socio-demographic; Perceived social status; Ethnic and racial background	MacArthur Scale of subjective Social Status; Center for Epidemiological Studies Depression Scale (CES-D)	Brief Pain Inventory (BPI)	*N* = 105 (completed)
Bartley et al., 2019 [35]	Quantitative *Cross-sectional study*	To empirically identify domains of resilience based upon psychological, social, and health-related factors and using cluster analysis, explore whether resiliency phenotypes differ across measures of physical function, pain intensity, disability, and psychological functioning	Socio-demographic; Predictors of Multisystem Resilience; Social functioning; Health comorbidities; Smoking status	Positive and Negative Affect Schedule (PANAS); The Adult Dispositional Hope Scale (ADHS); PROMIS Positive Affect and Well-Being Scale; Life-Orientation Test-Revised (LOT-R); PROMIS support (emotional, instrumental, informational); Anthropometric tests (Healthometer, BMI); Health comorbidities; Smoking status	Functional performance and movement-evoked pain (MEP) were measured using the Back Performance Scale (BPS); Short form of the PROMIS Physical Function; PROMIS Pain Intensity scale; Roland-Morris Disability Questionnaire (RMDQ); PROMIS depression scale; Brief resilience scale; World health organization quality of life-brief (WHOQOL-BREF)	*N* = 69 (completed)
Bernier Carney et al., 2021 [36]	Quantitative *Prospective cohort study*	To explore the degree to which increased psychological stress, self-reported pain scores, and pain sensitivity (primary risk factors) during a phase of acute LBP are independently predictive of pain trajectories over time; and to identify if these primary risk factors, taken together at baseline, present a model predicting the trajectory of low back pain	Socio-demographic; BMI and smoking status; Perceived psychological stress	Perceived Stress Scale (PSS);	Quantitative sensory testing (QST); Brief Pain Inventory-Short Form (BPI)	*N* = 217 (completed)
Boissoneault et al., 2017 [37]	Qualitative *Narrative review*	To consider the strengths and limitations of existing literature and propose suggestions that may lead to the development of parsimonious, cost-effective, and accurate predictive models of LBP chronicity	Socio-demographic; General health characteristics; Occupational factors	Pain Resilience Scale; Optimal Screening for Prediction of Referral and Outcome cohort yellow flag assessment tool (OSPRO-YF); Pain Self-Efficacy Questionnaire short form; Health behaviors: Alcohol Use Disorders Identification Test, Pittsburgh Sleep Quality Index, Örebro Musculoskeletal Pain Screening Questionnaire (ÖMPSQ);	Multiple outcomes related to chronicity, including functional disability, LBP-related work absence, pain intensity,and pain absence/presence; Roland-Morris Disability Questionnaire (RMDQ); Impact of LBP on productivity ought to prioritize return to work when defining recovery	NA
Buruck et al., 2019 [38]	Quantitative *Systematic review w/wo Meta-analysis*	To synthesize the evidence on the potential relationship between psychosocial work factors from the Areas of Worklife (AW) model (workload, job control, social support, reward, fairness, and values) and chronic low back pain (CLBP; unspecific pain in the lumbar region lasting 3 months or longer)	Psychosocial risk factors included in the Areas of Worklife model	Self-report measures of psychosocial job exposure: combined index of job control, decision authority, social support (combined, from colleages, from supervisor)	CLBP: unspecific pain in the lumbar region lasting 3 months or longer	*N* = 18 (included studies) *N* = 19,572 (completed)
Campbell et al., 2013 [39]	Quantitative *Prospective cohort study*	To investigate, in patients with LBP consulting in a primary care setting, which prognostic factors predict poor pain and disability outcomes 5 years later and to compare these with predictors of earlier short-term outcomes at 6-month follow-up in the same cohort	Demographic predictors; Behavioral coping and participants’ belief’s and confidence in their ability; Occupational predictors	The Illness Perception Questionnaire-Revised (IPQ-R); The Coping Strategies Questionnaire 24 (CSQ-24); The Pain Self-Efficacy Questionnaire (PSEQ);	Chronic Pain Grade (CPG)	*N* = 1591 (recruited) *N* = 488 (completed)
Chau et al., 2023 [40]	Mixed-method *Consortium of subject-matter experts and Umbrella review*	Summarizes the initial work of the Theoretical Model Working Group. It includes a three-stage integration of expert opinion and an umbrella literature review of factors that affect cLBP severity and chronicity	Socio-demographic; Social and cultural environnement; Occupational factors; Personnal/medical History; Perceived stress	Social context (perceived support; financial strain) Personal history (ACEs; PTSD, discrimination, perceived stress); Societal Input; Social Context (SES, Perceived support, financial strain)	cLBP outcomes or measures of patients’ cLBP experiences	*N* = 45 (included studies)
Chou et al., 2018 [41]	Qualitative *Qualitative evidence synthesis*	What needs of non-biomedical services are perceived by people with low back pain?	Needs for: Work and return to work; Financial support; Assistive devices and assistance with the home environment; Psychosocial support	Participant-perceived needs related to: occupation and return to work; financial support; Social support; assistive devices and assistance within the home environment	Duration of LBP	*N* = 20 (included studies) *N* = 522 (completed)
Darlow et al., 2013 [42]	Qualitative *Semistructured qualitative interview*	To understand why people have particular beliefs about low back pain and the impact of these beliefs To explore the relationship between patients’ and clinicians’ beliefs	Relationship between patients’ and clinicians’ beliefs	Influence of beliefs assessment	Duration of LBP	*N* = 23 (completed)
Draper-Rodi et al., 2018 [43]	Qualitative *Qualitative evidence synthesis*	The aims of this scoping review were to: 1. Summarise current guidance for the assessment and prognosis of NSLBP and the BPS model, 2. Inform the development of a training programme synthesising evidence for the evaluation of NSLBP in a BPS context	Socio-demographic; Occupational factors; Relationships at work; Social support; Influence of clinicians	NA	NA	*N* = 41 (included papers)
Ferreira et al., 2010 [44]	Quantitative *Systematic review w/wo Meta-analysis*	To identify factors associated with health care-seeking from studies with well-defined groups of care-seekers and non-seekers with non-specific LBP	Socio-demographic; Social functioning; Occupational factors; Social climate; BMI and smoking status	Care-seeking determinant	Seekers and non-seekers of care	*N* = 11 (included studies) *N* = 13,486 (completed)
Gomes et al., 2023 [45]	Quantitative *Prospective cohort study*	To investigate the presence of different courses of pain across five years in a sample of patients with active CLBP; to examine their cumulative impact on disability and HRQoL and; to identify prognostic indicators for the persistent LBP course	Socio-demographic; Lifestyle; Health-related indicators; Psychosocial	Health-Related Quality of Life (EQ-5D-3L)	Portuguese version of the Health Assessment Questionnaire (HAQ)	*N* = 1201 (recruited) *N* = 634 (completed)
Gray et al., 2011 [46]	Quantitative *Systematic review w/wo Meta-analysis*	To evaluate the content, psychometric properties and clinical feasibility of instruments available for the assessment of work-related psychosocial factors (Blue Flags) in working age adults with NSLBP. This will provide clinicians with evidence based information upon which to guide their decision making	Blue flags construct; Individual’s perceptions about work	Back Disability Risk Questionnaire (BDRQ); Occupational Role Questionnaire (ORQ); Obstacles to Return to Work Questionnaire (ORTWQ); Psychosocial Aspects of Work Questionnaire (PAWQ); Vermont Disability Prediction Questionnaire (VDPQ); Modified Work APGAR (MWA)	NA	*N* = 5630 (completed)
Grotle et al., 2010 [47]	Quantitative *Prospective cohort study*	To compare the contribution of physical, psychological and social indicators to predicting disability after one year between consulters with LBP of less than 3 months duration and more than 3 months duration	Socio-demographic; Work status and work dissatisfaction; Coping strategies	The Coping Strategies Questionnaire 24 (CSQ-24)	Self-reported disability due to LBP, measured by the RMDQ	*N* = 2526 (completed)
Hayden et al., 2019 [48]	Quantitative *Systematic review w/wo Meta-analysis*	To synthesise evidence on the association between recovery expectations and disability outcomes in adults with low back pain, and explore sources of heterogeneity	Individual expectations related to futur low back pain outcome	Individual recovery expecations: general, self-efficacy and treatment expectation	Work participation; Roland-Morris Disability Questionnaire (RMDQ) or the Oswestry Disability Index (ODI); Pain intensity: Visual analogue scale (VAS) or numerical rating scale (NRS)	*N* = 60 (included studies) *N* = 30,530 (completed)
Heymans et al., 2010 [49]	Quantitative *Prospective cohort study*	To investigate the prognostic value of demographic, work, clinical, and psychosocial variables. Another objective was to introduce and investigate the prognostic value of new variables a clinically important change in pain intensity and disability status on the development of chronic LBP	Socio-demographic; Lifestyle; Work related psychosocial variables	Baecke Physical Activity Questionnaire (BPAQ); Dutch Musculoskeletal Questionnaire; Dutch version of the Job Content Questionnaire; Questionnaire concerning job task satisfaction; Pain Coping Inventory Questionnaire	Pain intensity: Numerical Rating Scale (NRS); Roland Morris Disability Questionnaire (RMDQ)	*N* = 629 (recruited) *N* = 628 (completed)
Hopayian et al., 2014 [50]	Qualitative *Qualitative evidence synthesis*	To describe the experience of health care of LBP and sciatica patients and the sources of satisfaction or dissatisfaction. A secondary aim was to describe the experience and satisfaction of patients who do not receive a diagnosis	Patient’s experience of healthcare	Process and content of care; Outcome of care; Patient-practitioner relationship and interpersonal skills; Treated as an individual/personalised care; Vital to have diagnosis; Service matters: access, appointment, cost; Legitimation: having a diagnosis means being believed	NA	*N* = 28 (included studies)
Iversen et al., 2015 [51]	Quantitative *Prospective cohort study*	To identify clinically relevant predictors for outcome among patients with chronic radiculopathy	Socio-demographic; Smoking status; Fear avoidance belief about work	Fear Avoidance Belief Questionnaire (FABQ)	Oswestry Disability Index (ODI); Visual Analogic Scale: back and leg pain	*N* = 116 (completed)
Jackson et al., 2022 [52]	Quantitative *Cross-sectional study*	To compare the Area Deprivation Index (ADI) and other measures of Socioeconomic Status (SES) in their ability to predict pain severity/interference	Socio-demographic; Self-identified race; Objective and subjective Socio-Economic Status	Area Deprivation Index (ADI); MacArthur Ladder of Subjective Social Status	Brief Pain Inventory – Short Form (BPI)	*N* = 129 (recruited) *N* = 104 (completed)
Járomi et al., 2021 [53]	Quantitative *Cross-sectional study*	To assess the quality of life (HRQoL) of nscLBP syndrome patients and the functional status of the spine, testing the validity of the HRQoL scale and comparing the scales with healthy adults’s mean values. We hypothesized that the socio-demographic parameters, self-rated physical disability (RMDQ- Roland Morris Disability Questionnaire) and low back pain specific knowledge (LBPKQ) would determine significantly the HRQoL measures by SF-36of nscLBP patients. This study aims help the better understanding of the disease and encourage health care workers to considering socio-demographic and knowledge factors also in planning interventions	Socio-demographic; Socio-environmental; Occupational factors; Disease-specific knowledge	Quality of Life (SF-36); Low Back Pain knowledge questionnaire	Roland Morris Disability Questionnaire (RMDQ)	N = 1155 (completed)
Karran et al., 2020 [54]	Mixed-method *Systematic review w/wo Meta-analysis and narrative review*	To determine, through systematic review and evidence synthesis, the current evidence concerning the relationships between SDH and the frequency or severity of CLBP To determine the associations between the SDH and CLBP-related disability, work absenteeism, and opioid prescription, because these are key contributors to the personal and societal burden of LBP	Social Determinants of Health	Identification of Social Determinants of Health guided by the PROGRESS Framework from the WHO	Frequency or severity (intensity) of cLBP	*N* = 2,161,617 (completed)
Karran et al., 2022 [55]	Mixed-method *Prospective cohort study and Open-ended qualitative interview*	To investigate the relationships between the SDH and health outcomes, to explore healthcare experiences, and to explore perceived barriers and facilitators to receiving optimal pain care	Socio-demographic; Social Determinants of Health individual and macro level indicators; Social disadvantaged experience of healthcare	Social History Screening (SHS Tool); Perceived Social Isolation – friendship Scale; Index of Relative Socioeconomic Advantage and Disadvantage (IRSAD); Modified Monash Model (MMM)	Health Outcomes: Pain intensity and pain interference: West Haven-Yale Multidimensional Pain Inventory (WHYMPI); Quality of Life: World Health Organization Quality Of Life Assessment (WHOQOL-Bref); Psychological Distress: Kessler-10 (K-10); Comorbidities: Functional Comorbidity Index (FCI)	N = 181 (completed) *N* = *164* *(quantitative study)* *N* = *17* *(qualitative study)*
Koenig et al., 2014 [56]	Quantitative *Observational longitudinal study*	To examine the relationship between biopsychosocial functioning and pain severity and to evaluate whether pain self-efficacy (PSE) mediates this relationship	Socio-demographic; Biopsychosocial functioning; Pain Self-efficacy	Quality of Life (SF-36); Pain Self-Efficacy Questionnaire (PSEQ)	Pain severity: the Short-Form McGill Pain Questionnaires (SF-MPQ)	*N* = 114 (recruited) *N* = 99 (completed)
Melloh et al., 2011 [57]	Quantitative *Prospective cohort study*	To identify factors that influence the progression of acute LBP to the persistent state at an early stage	Working conditions; Quality of life; Maladaptative cognitions	Index 1 ‘Working conditions’; Index 2 ‘Depression and maladaptive cognitions’; Index 3 ‘Pain and quality of life’	Oswestry Disability Index (ODI)	*N* = 53 (completed)
Nieminen et al.,2021 [58]	Quantitative *Systematic review w/wo Meta-analysis*	To identify the prognostic factors for pain chronicity in patients with LBP and to provide an update on the existing data	Socio-demographic; Personal factors and medical history; Support at home and at work; Psychosocial factors	NA	The main outcome was nonspecific CLBP with or without pain radiation, but specific nerve root disorders were excluded	*N* = 25 (included studies)
Nordeman et al., 2017 [59]	Quantitative *Prospective cohort study*	To assess if body function, activity, participation, health-related quality of life and lifestyle behavioural factors can predict activity limitation in women with chronic low back pain (CLBP) in primary healthcare (PHC) 2 years later	Socio-demographic; Lifestyle behavioural factors; Work participation; Environmental factors; Health-related quality of life	Social Support Survey (MOS-SSS); Quality of Life (SF-36); Physical component and mental component summary (PCS/MCS)	Roland Morris Disability Questionnaire (RMDQ)	*N* = 130 (completed)
Otero-Ketterer et al., 2022 [28]	Quantitative *Systematic review w/wo Meta-analysis*	To display an up-to-date overview of high-level research evidence providing longitudinal data on biopsychosocial prognostic factors of outcomes in individuals with non-specific low back pain	Socio-demographic; Occupational factors; Patient experience	Fear Avoidance Belief’s Questionnaire (FABQ); Self-reported Work physical demands	The International Classification of Functioning, Disability and Health (ICF) framework — pain intensity, functional status and work participation and recovery	*N* = 257,208 (completed)
Pincus et al., 2013 [60]	Qualitative *Narrative review*	To evaluate the utility of the model in reference to rising rates of back pain-related disability, by identifying the most promising avenues for future research in biological, psychological, and social approaches, promising combinations of all 3 approaches, and obstacles to effective implementation of biopsychosocial-based research and clinical practice	Social and organizational factors influence; Occupational factors;Financial support	Factors operating both at an individual (individuals' status, individuals' perception and reaction to their status) and at a group level (regional or national level, the process at group level)	NA	NA
Preuper et al., 2011 [61]	Quantitative *Cross-sectional study*	To confirm or refute the finding that a strong relationship exists between psychosocial distress and self-reported disability in patients with nonspecific chronic low back pain (CLBP) by analyzing this relationship in patients with CLBP admitted for treatment in six RCs	Socio-demographic; Psychosocial distress; Social security benefits	Symptom Checklist-90-Revised (SCL-90-R); Global Severity Index (GSI)	Roland Morris Disability Questionnaire (RMDQ);	*N* = 293 (completed)
Rabey et al., 2017 [62]	Quantitative *Prospective cohort study*	To derive prognostic models from both individual factors and subgroup categorization at 1-year follow-up in a large CLBP cohort	Socio -demographic; Health dimension; Lifestyle dimension; Occupational factors; Social support; Psychosocial	General health (COOP/WONCA charts); Physical Activity Questionnaire (IPAQ); West Haven-Yale Multidimensional Pain Inventory (WHYMPI)	Pain intensity (NRS); Disability (RMDQ); CLBP duration; Global Rating of Change (NRS); Bothersomeness (NRS)	*N* = 294 (completed)
Ramond et al., 2011 [63]	Quantitative *Systematic review w/wo Meta-analysis*	To review the evidence on the prognostic value of psychosocial factors on transition from acute to chronic non-specific LBP in the adult general population	Socio -demographic; General health patient’s expectations; Occupational factors; Social support; Cognitive and behavioural factors; Compensation issues; Care provider’s judgement	Duke-UNC-functional social support; General Health Questionnaire (GHQ) social dysfunction; Quality of Life (SF-36); Modified Work APGAR (MWA)	Patient-centered outcome criteria—LBP outcome: Pain; Disability; Work status; Participation; Patient satisfaction; Mixed criteria	*N* = 23 (included studies)
Shaw et al., 2013 [64]	Qualitative *Narrative review*	NA	Socio-demographic; Characteristics of the psychosocial environment at home, clinic and the workplace	Blue flags (subjective perceptions and beliefs about work): Obstacles to Return to Work Questionnaire (ORQ); Job Content Questionnaire; Black flags (factual circumstances surrounding work) Work Organisation Assessment Questionnaire (WOAQ)	NA	NA
Tagliaferri et al., 2020 [65]	Qualitative *Narrative review*	To discuss the various factors that may influence pain intensity at the level of the central nervous system (CNS) To review the multiple domains affected in individuals with CLBP To propose outcome measures that capture each domain and highlight treatments that may impact these measures	Socio-demographic; Occupational factors; Health-related Quality of Life; Social functioning	Social Functioning; Work Absenteeism; Health-Related Quality of Life	Biological and Functional Outcomes; Psychological Outcomes;Social Outcomes	NA
Wilkens et al., 2013 [66]	Quantitative *Prospective cohort study*	To identify predictors of 1-year disability status in a group of CLBP patients with degenerative findings, visualized on MRI, who were included in a RCT comparing glucosamine with a placebo, where no differences between groups were observed	Socio-demographic; BMI and smoking status; Psychosocial and Quality of Life	Fear Avoidance Belief Questionnaire (FABQ); Quality of Life (EQ-5D)	Norwegian version of Roland Morris Disability Questionnaire (RMDQ); Low back and leg pain (Numerical rating scales)	*N* = 250 (completed)
Wippert et al., 2022 [67]	Quantitative *Observational longitudinal study*	Analyze the associations between different types of stress with non-specific current LBP and its influence on the development of non-specific CLBP as well as on fatigue and depressive mood (as individual outcomes or as symptom triad) within 1 year. Identify the most important stress types regarding the development of non-specific chronic LBP, fatigue, and depressive mood within 1 year. Identify the most predictive stress related (neuro) pattern set regarding the development of non-specific CLBP within 1 year and to test its accuracy for diagnostics	Socio-demographic; Lifestyle; Life changing events; Perceived stress and chronic stress	Trier Inventory for Chronic Stress (TICS); Perceived Stress Scale (PSS); Effort-Reward-Imbalance Questionnaire; Inventory of life-changing events	Chronic Pain Grade (CPG)	*N* = 140 (completed)

*NA* Not Apllicable/Not relevant

**Table 3:** Social domains and factors categories identified in the included articles

**Socio-demographic**
**Age and gender**
**Civil status**
**Place of living**
**Ethnicity**
**Education level**
**Socio-Economic Status** (SES): Social class; higher (managerial/professional and intermediate occupations, and self-employed) and lower (lower supervisory/technical, semi-routine and routine occupations); income; access to care; Socio Economic Classification (SEC): level of income; distinction between blue-collar and white-collar workers; Area Deprivation Index (ADI): a validated objective measure of SES at the area level, using neighborhood disadvantage
**Socio-occupational**
**Work status**: employed/not employed; work absence due to LBP/currently employed in normal job/currently employed on reduced duties because of LBP/currently unemployed because of LBP/currently unemployed because of other reasons; Compensation status
**Employment form**: job-related physical variable (sedentary work with less that 30 min physical activity/sedentary work with more than 30 min physical activity/light physical labour/heavy physical labour); Higher physical work demands/Sedentary work and desk/chair set up/Higher work demands and/or history of specific manual work (including farming, nursing, truck driving)/Sedentary work
**Working conditions**: job satisfaction, work attitudes; social workplace support; job insecurity; concentration requirements; work organisational problems and interruptions; time pressure as well as physical and emotional stress; lack of social support as well as lack of method control and lack of time control. Blue Flags are defined as the individual’s perceptions about work, whether accurate or inaccurate, that can affect disability. Blue Flag constructs include, for example: negative expectations of return to work; job dissatisfaction; stress at work; work-related fear avoidance beliefs; perceptions of physical job demands; and poor colleague or supervisor relationships. Psychosocial risk factors included in the Areas of Worklife (AW) model (workload, job control, social support, reward, values or fairness)
**Social support at work**: consists of supervisor support and coworker support
**Socio-environmental**
**Environmental and social support**: Medical Outcome Study Social Support Survey registered private social support: emotional-informational, tangible, affectionate support and positive social interaction. Social environment includes social support and invalidating, stigmatizing, or discriminating responses from others/Spouse and family behaviour and/or negative expectations of recovery. Social factors at home
**Social Determinants of Health** (SDH): were organized according to the PROGRESS framework, which grouped the SDH into 8 domains: Place of residence; Race, ethnicity, culture and language; Occupation; Gender and sex; Religion; Education; Socioeconomic status; and Social capital; Individual-level indicators: Social history screening; Perceived social isolation/SDH, macro-level indicators: Index of social deprivation (summarizes information about the economic and social conditions of people and households within an area); Index of remotness
**Social functioning:** is considered as the individual’s ability to engage in social activities/social identity of the individual/perceptions of physical health and level of social interaction; social environment/social impairment/functional impairments
**Financial support: **financial instability/financial strain/social security benefits
**Coping strategies**: can include religious or spiritual practices, like meditation and prayer, that are incorporated as a cognitive process of self-management
**Subjective social status**: The MacArthur Scale of Subjective Social Status is a commonly used measure of subjective social status that assesses an individual’s sense of social status across the socioeconomic status indicators
**Global health data**
**Health status**: presence of differing types of comorbidities/general health, BMI, pharmacological treatment
**Lifestyle**: physical activity; alcohol consumption; smoking status; sleep quality and quantity
**Health-related indicators:** disability and quality of life; well being
**Personal history**: Substance use; trauma; ACEs (ACE: Agreeableness, Conscientiousness, Extraversion); number and load critical life events/Stressful life events
**Psycho-social**
**Chronic and perceived stress**: Perceived stress; Stress burden; Stress symptoms (include stress at home and at work)
**Psychological constructs***:* Mental well-being; Depression; Anxiety; Post-traumatic Stress Disorder; Antisocial personality disorder; Any psychiatric diagnosis; Somatization; Fear avoidance belief; Perceived risk of persistence; Catastrophizing; Low tolerance of pain; Coping by ignoring pain; Predictors of Multisystem Resilience; Psychological Attributes; Psychological emotional; Psychological cognitive; emotional distress *(depression and maladaptive cognitions: contained depression, somatization, resigned attitude towards the job, fear-avoidance, rumination, helplessness, catastrophizing and negative expectations on return to work. Pain Beliefs/Affective State/Personality Traits)*
**Individual recovery expectations**: general expectations and treatment expectations; General health Patient’s expectations; Patient expectation of a “techno-fix”
**Pain Self-Efficacy**: the patient’s confidence in performing daily activities despite experiencing pain, tolerating pain and coping with pain; two aspects of hope: pathways and agency
**Patient’s experiences of health care**
**Disease-specific knowledge**
**Patients’ perceived needs of non-biomedical services for LBP**: The term “non-biomedical services” was used to incorporate a variety of services for non-biomedical determinants of health, such as: environmental factors, social factors, community factors, socioeconomic factors, and health behaviours. Needs related to occupation and return to work; financial support; assistive devices and assistance with the home environment; psychosocial support
**Clinicians’ Influence:** For exeample Care provider’s judgement
**Patient-reported experience**: Process and content of care; Outcome of care Recognizing the expert; Patient-practitioner relationship and interpersonal skills; Treated as an individual; personalised care; Information; Vital to have diagnosis; Service matters; Legitimation. Lack of satisfaction with previous treatment for back pain; Experience of conflicting diagnoses or explanations for back pain; Use of diagnostic language; A careful initial examination; Advice from healthcare professional to withdraw from work; Labeling patients with Yellow Flags and sanctioning disability

The Six Social Domains (underlined) that emerged from further analysis of the 28 factor categories (bold font)

### The socio-demographic domain

The factors in this domain are widely studied in epidemiology and social sciences to characterize populations and identify disparities. In research, they are also used as adjustment variables to control for confounding. In the context of CLBP, they help understand social health inequalities and identify at-risk populations. These include: age and gender (defined in a binary manner) (*n* = 26); education level (*n* = 17); civil status (*n* = 8), place of living (*n* = 3), ethnicity (*n* = 7). The SD domain also includes data related to professional class, socio-economic status or social class (*n* = 12). As shown in Table 3, the socio-economic status factor category contains numerous variables, including annual household income, professional status, and the distinction between blue-collar and white-collar workers, as well as salaried and non-salaried workers [40, 69], and includes both objective and subjective measures. For example, The Area Deprivation Index is a validated objective measure of the socio-economic level of a geographical area [58].

### The socio-occupational domain

Factor categories in this domain were frequently investigated, albeit slightly less often than those in the SD domain. These factors relate to an individual's work environment as well as to employment as a structuring institution and a key identity marker in adulthood. These include: work status (*n* = 19), which refers to whether individuals are employed or unemployed, on sick leave, or working part-time due to CLBP; factors related to the employment form and the physical characteristics of the work (*n* = 12), among which sedentary work, physical constraints, and manual work are contributors to CLBP; psychosocial factors linked to working conditions (*n* = 15), such as job satisfaction, attitudes to work, social support from colleagues and supervisors, job insecurity, required concentration, lack of agency or social recognition, pressure to perform, chronic worrying and organizational issues [63, 73]. Gray et al. [52] investigated blue flags, related to individuals' perceptions about work. Blue flag constructs include, for example, negative return-to-work expectations, job dissatisfaction, stress at work, work-related fear-avoidance beliefs, perceived physical demands of work and poor relationships with colleagues or supervisors. Buruck et al. [44] explored the psychosocial risk factors included in the Areas of Worklife model (workload, job control, social support, reward, values or fairness). Finally, six studies examined the social support and workplace climate.

### The socio-environmental domain

This domain encompasses factors related to an individual's social networks and community structures that shape their social environment. These elements can provide support at different levels: emotional, informational, or instrumental. They influence not only psychological and social well-being but also access to various essential resources. The most frequently investigated factor categories in this domain were environmental and social support (*n* = 11). Private social support, which can be assessed using various tools including the Medical Outcome Study Social Support Survey (MOS-SSS) that measures emotional-informational, tangible, affective support, and positive social interactions [65]. Characteristics of the social environment also include disabling, stigmatizing or discriminating responses from others, spousal and family behaviour, as well as negative recovery expectations [46]. The SE domain also contains social functioning (*n* = 4), defined as the individual's ability to engage in social activities, social identity and level of social interaction [71]. Financial support or financial instability (*n* = 7) contributes to the modulation and development of CLBP, as well as stress levels. According to Preuper et al. [67], patients experiencing financial instability face difficulties in accessing healthcare services, which can lead to a deterioration of their condition and the maintenance of pain. Additionally, Chou et al. [47] highlight that economic constraints limit patients’ ability to follow treatment recommendations. Within the SE domain, coping strategies (*n* = 2), including religious practices [46], are also explored. Subjective social status (*n* = 2) is another studied factor that may mediate the development and persistence of CLBP, assessed using the MacArthur Scale of Subjective Social Status [40, 58]. Finally, Social Determinants of Health (SDH) (*n* = 2) were investigated using the PROGRESS conceptual framework [60], which groups SDH into eight domains: place of residence, race, ethnicity, culture and language, occupation, gender and sex, religion, education, socio-economic status and social capital. SDH were also investigated by individual-level indicators (social history screening, perception of social isolation) and macro-level indicators (Social Deprivation Index and Remoteness Index) [61]. The inclusion of these factors highlights the potential importance of networks and social support in mitigating pain effects and preventing psychological stress [61].

### The global health data domain

This domain includes four factor categories that share common characteristics. Although often presented as purely biological or explained in terms of individual behaviour, we have included them in this domain due to their strong grounding in social and structural contexts. This classification reflects their inherent social dimension within the BPS model, where they are not reducible to a purely biological explanation but instead lie at the intersection of several dimensions. Health status (n = 9) includes co-morbidities, medical history and body mass index. Obesity (or high body mass index) has been identified as a risk factor for the persistence of low back pain and disability [64, 72]. Obesity could also be included in the category we have called lifestyle (*n* = 16), which encompasses people's health behaviours and includes physical activity, alcohol and cigarette consumption, as explored by Nordeman [65], and sleep quality and quantity. Tagliaferri [71] describes the correlation between sleep quality and the development of CLBP and disability. Other authors categorise some factors as health-related indicators (*n* = 13), describing health through quality of life, disability and well-being. A final factor category in this social domain is personal history (*n* = 4) which includes substance use (other than alcohol and cigarettes), critical or stressful life events and past trauma.

### The psycho-social domain

Although the factor categories in this domain tend to be classified as psychological, their development and influence are inseparable from social contexts and from each individual’s unique living conditions. They can be understood from an individual, family, or professional perspective, as well as from broader social patterns that affect groups of people. Their inclusion in this domain highlights their intrinsically social character within the BPS model and emphasises that they cannot be reduced to a purely psychological explanation. A large number of studies (*n* = 22) explore the Psychological Dimension factor category using psychological constructs such as mental well-being, post-traumatic stress, fear avoidance, catastrophizing and personality traits, as well as depression and anxiety that may influence disability and recovery time [69]. For example, Aroke and colleagues [40] highlight a mediating effect of depression between perceived social status and pain severity in people suffering from CLBP. The different dimensions of chronic stress and the notion of perceived stress (*n* = 5) are also discussed, for example as a mediator linking household income and pain persistence [42]. Individual expectations of recovery (*n* = 4) appear to be associated with better participation in work [54]. Finally, the notion of self-efficacy (*n* = 4) is influenced by the environment and social support is associated with CLBP at 5 years [45].

### The patient’s experience of health care domain

The factor categories we attributed to this social domain were mainly identified in qualitative studies. They reflect individual experiences, which when combined make it possible to highlight the influence of social structures, such as institutions, norms and social roles [47], as well as power relations, including the influence of clinicians on patients (n = 6). For example, the influence of professional judgement and legitimisation through diagnosis [56, 69] or clinicians’ influence on patients’ expectations of recovery [48]. Patient-reported experience of care (n = 4) includes situations such as the lived experience of healthcare for socially disadvantaged individuals suffering from CLBP [61]. It also includes the experience of contradictory diagnoses, the use of “diagnostic” language, dissatisfaction with care, and the therapeutic relationship [49, 56]. Chou [47] investigated patients’ perceived non-medical service needs (n = 1), which include environmental, social, community, socio-economic and health behaviour factors. They highlight non-medical needs that could lead to more favourable outcomes for CLBP, such as the need for financial support, psychological support, assistive devices, and assistance with the home environment. Finally, Járomi [59] studied patients’ disease-related knowledge (n = 1) and observed a correlation between the influence of health literacy specific to low back pain and quality of life—considered as a relevant outcome related to CLBP.

**Fig. 2:**
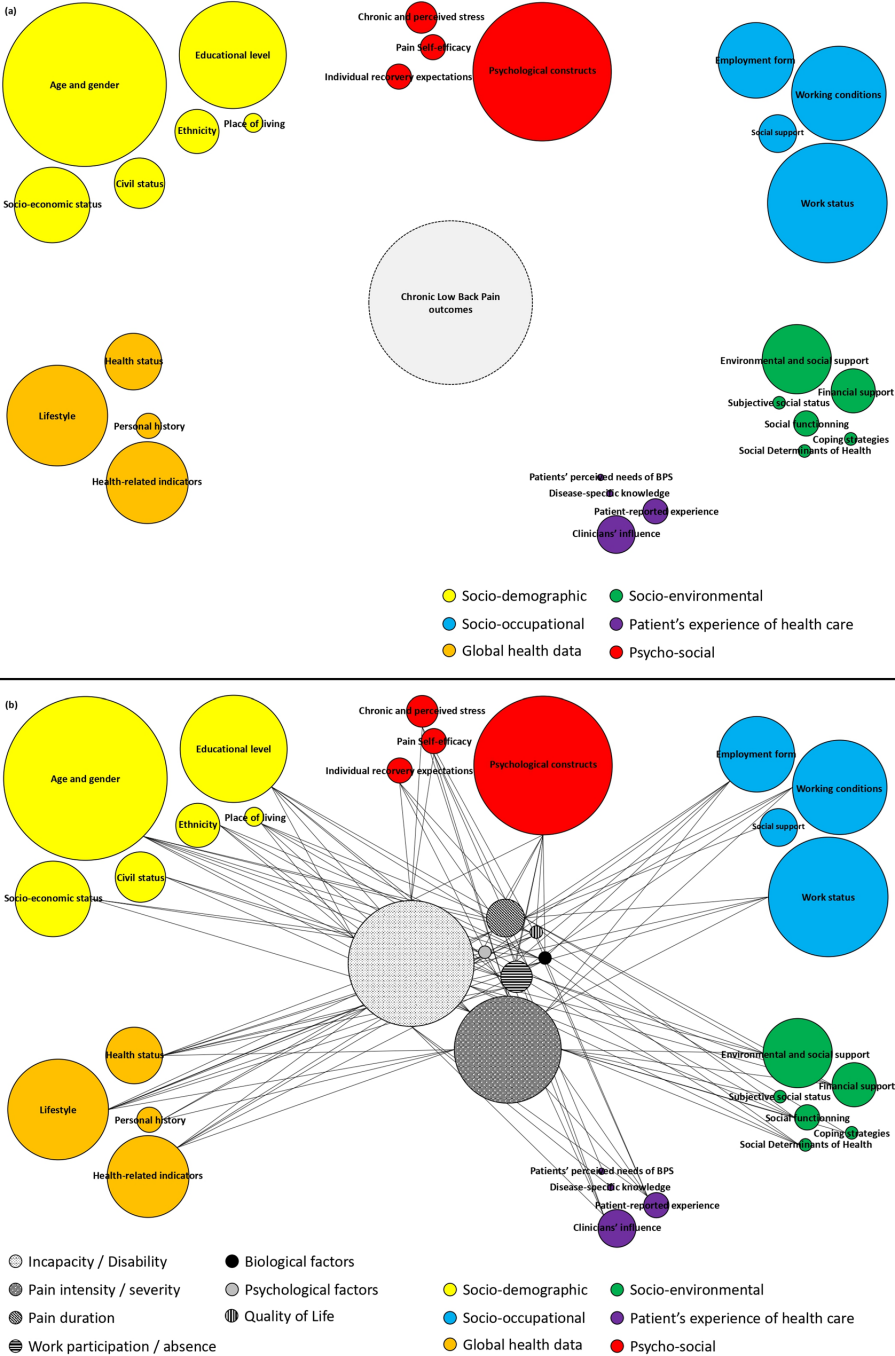
**a** Domains and Categories of social factors. Sphere size indicates the frequency with which that factor category was assessed. Sphere colours correspond to the six social domains. **b** Domains and Categories of social factors. The most commonly used CLBP outcomes in the centre, represented by grey spheres. The size of the sphere reflects the frequency of use of an outcome. The lines indicate that the influence between a given outcome and a given factor category has been evaluated

Table 4 and Fig. 2a show the number of articles that studied each factor category. These show that not all factor categories are investigated with the same frequency and that certain social domains (e.g. PEHC) are underrepresented. The lines in Fig. 2b represent studies that investigated the impact of each factor category on the seven CLBP outcomes identified in the included articles. Each experimental study or literature review examined the influence of a variety of social factors on different CLBP outcomes, primarily pain intensity (*n* = 17) and disability (*n* = 20) (Roland-Morris Disability Questionnaire, Oswestry Disability Index). Other outcomes included pain duration (*n* = 6) and work participation (*n* = 5) (work absences). A small number of studies used other outcomes such as quality of life [41, 61], demand for care and services [47, 50], psychological distress [61], co-morbidities or patient satisfaction [69].

**Table 4:** Detailed breakdown of which studies investigated each of the 28 factor categories

× *: factor with the prognostic value studied × *: factor without the prognostic value studied*	**Socio-demographic**						**Socio-occupational**				**Socio-environmental**						**Global health data**				**Psycho-social**				**Patient’s experience of health care**			
	Age and gender	Civil status	Place of living	Ethnicity	Education level	Socio-economic status	Work status	Employment form	Working conditions	Social support at work	Environmental and social support	Social Determinants of Health (according to the PROGRESS)	Social functionning	Financial support	Coping strategies	Subjective social status	Health status	Lifestyle	Health-related indicators	Personal history	Chronic and perceived stress	Psychological constructs	Individual recovery expectations	Pain Self-Efficacy	Disease-specific knowledge	Patients’ perceived needs of non-biomedical services for LBP	Clinicians’ Influence	Patient-reported experience
Aroke et al., 2020 [34]	× *			× *	× *	× *	× *									× *	×					×						
Bartley et al., 2019 [35]	× *	× *		× *	× *	× *	× *						× *				× *	× *				× *						
Bernier Carney et al., 2021 [36]	× *			×		× *		×							×			×	×		× *	×						
Boissoneault et al., 2017 [37]	×						×				×						×	×				×						
Buruck et al., 2019 [38]									× *	× *																		
Campbell et al., 2013 [39]	× *				× *	× *	× *															× *		× *				
Chau et al., 2023 [40]	× *	×		× *	× *	× *	× *	× *	× *	× *	× *			×	×			× *		×	× *	× *						
Chou et al., 2018 [41]									×		×			×								×				×		
Darlow et al., 2013 [42]																							×				×	
Draper-Rodi et al., 2018 [43]	× *					× *		× *	× *	× *	× *											× *		× *			× *	× *
Ferreira et al., 2010 [44]	×	×					×	×	×	×			×					×	×								×	×
Gomes et al., 2023 [45]	× *	× *			× *		× *	× *										× *	× *			× *						
Gray et al., 2011 [46]									×																			
Grotle et al., 2010 [47]	× *				× *	× *	× *		× *													× *						
Hayden et al., 2019 [48]																							× *					
Heymans et al., 2010 [49]	×							× *	× *									×				× *						
Hopayian et al., 2014 [50]																											×	×
Iversen et al., 2015 [51]	× *				× *		× *											× *				× *						
Jackson et al., 2022 [52]	×			×	× *	× *										× *												
Járomi et al., 2021 [53]	× *	× *	×		× *		×	× *																	× *			
Karran et al., 2020 [54]	× *		× *	× *	× *	× *	× *	× *			× *	× *		× *														
Karran et al., 2022 [55]	×		×		×		× *				× *	× *		× *			×	×	×		× *	×						×
Koenig et al., 2014 [56]	×												× *				× *		× *			× *		× *				
Melloh et al., 2011 [57]	× *							× *	× *									×	× *			× *						
Nieminen et al.,2021 [58]	× *				×	×	×	× *	× *	×	×			×			× *	× *	× *	× *		× *						
Nordeman et al., 2017 [59]	× *	×					× *				× *						× *	× *	× *		× *	× *						
Otero-Ketterer et al., 2022 [28]	×				× *			× *											× *			× *	× *	×				
Pincus et al., 2013 [60]							×	×	×					×														
Preuper et al., 2011 [61]	×	×			×		×							×					×			× *						
Rabey et al., 2017 [62]	× *				× *				× *		×						×	×	× *	×		× *						
Ramond et al., 2011 [63]		× *			× *	× *	× *		× *		× *											× *	× *				× *	
Shaw et al., 2013 [64]	×			×		×			×	×	×																×	
Tagliaferri et al., 2020 [65]							×						×					×	×									
Wilkens et al., 2013 [66]	× *				× *		× *										× *	× *	× *			× *						
Wippert et al., 2022 [67]	×								× *									×		× *	× *

### Citation network

**Fig. 3:**
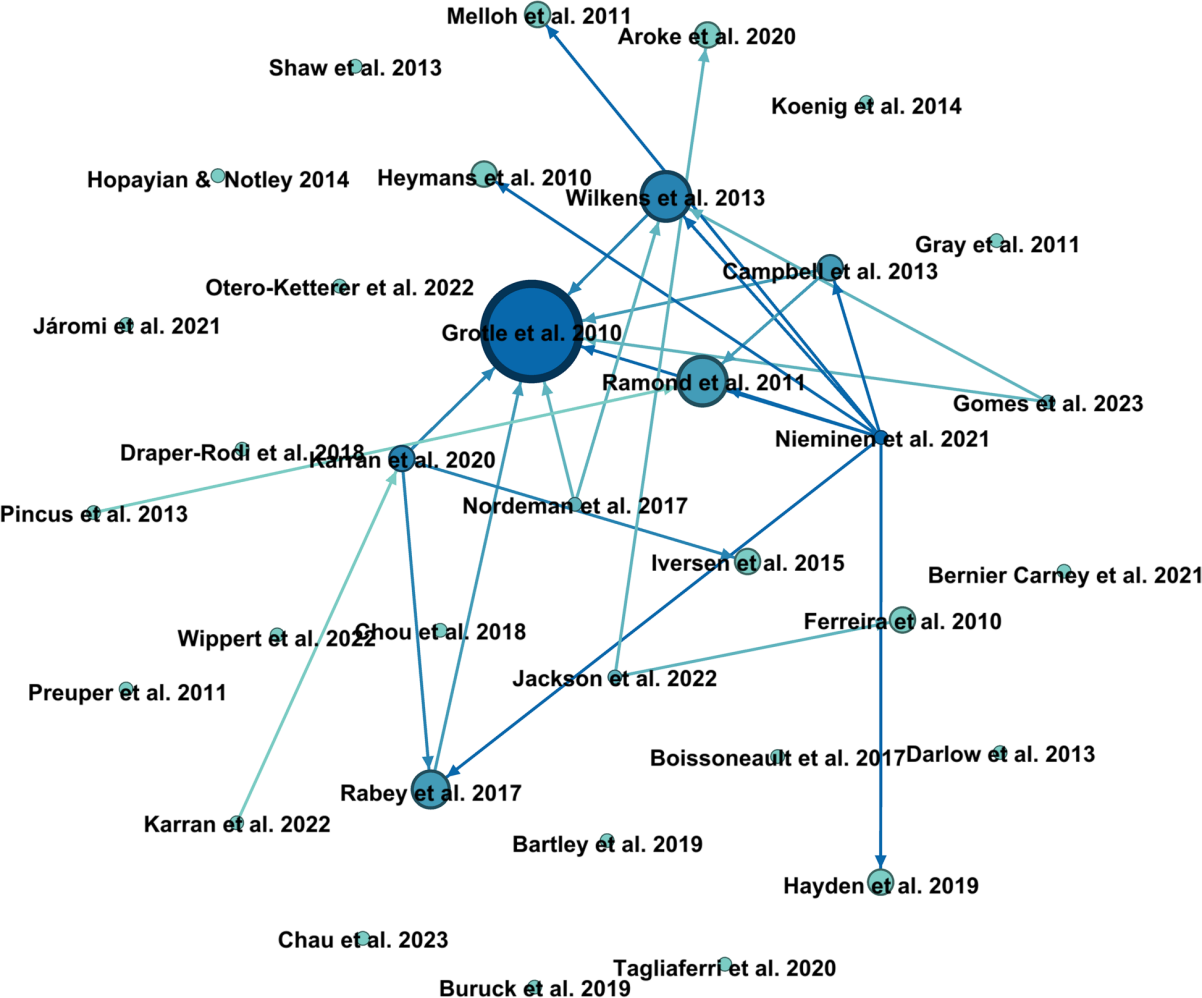
Citation network for the articles included in the scoping review. Each sphere represents an article, with its size proportional to the number of times it has been cited by other articles included in this review. The arrows indicate citation relationships, showing whether an article cites or is cited by another

Figure 3 illustrates the citation network, showing all citations between each of the 35 included articles. This shows that many articles are isolated, without citation links to any of the others. A single, relatively old article is, however, frequently cited [53]. This figure suggests that our selection encompasses a large range of literature about social factors.

## Discussion

The articles included in this scoping review, contained many different factors that we classified into 28 factors categories and 6 social domains. This classification method highlights both individual- and societal- level perspectives essential for understanding social factors, encompassing various levels of influence, including institutional, family, work-related, and environmental contexts. In line with our criteria, all of the included studies used a BPS model but we observed that while there might be a clear theoretical distinction between biological and PS factors, these two dimensions are often put together when considered at the level of the individual. This is in part because it can sometimes be complex to draw a clear boundary between the 3 domains of the BPS model. For example, some authors consider body mass index (BMI) as a comorbidity and analyse it from a biological perspective, while others view it as a social factor and classify it as socio-demographic or lifestyle data. Smoking status presents a similar issue. Thus, the inclusion of the GHD domain may appear debatable, as it encompasses general health indicators. However, we incorporated it into our framework because comorbidities and lifestyle factors are significantly influenced by social factors. Indeed, BMI is partially shaped by social determinants, particularly disparities in access to healthy food and sport facilities, which are themselves influenced by economic insecurity and education level. Furthermore, cultural and social norms significantly impact dietary behaviours and body image, with distinct influences based on gender and levels of social support [75–78]. Moreover, an elevated BMI is frequently associated with stigma and discrimination, particularly in healthcare settings, which can limit access to medical care and compromise the quality of treatment for individuals experiencing chronic pain [79, 80]. While BMI is widely acknowledged as a risk factor for the development of chronic pain [81, 82], it cannot be conceived of as merely a consequence of individual behaviours or as an isolated biological component. For those with CLBP, considering BMI within a broader BPS perspective allows for a better understanding of its impact on health and patient care pathways.

A similar rationale applies to sleep quality and tobacco consumption, which are closely linked to living conditions and individuals' social determinants, such as social relationships, working and living conditions (e.g., noise, insecurity, neighbourhood social ties) [83–87]. These factors play a significant role in the development and persistence of CLBP, further highlighting the importance of an approach that fully integrates social factors. Mardian et al. [88] suggest that this is because these factors are interconnected, with the human psyche and social relationships influencing and being influenced by biology. The absence of clear boundaries between factor categories was also found within the PS domain, as some articles reduced social factors to internal factors, specific to the individual [31, 54].

We observed significant variations in the conceptualisation of social factors, possibly resulting from an evolution in the focus on certain factors across time, or from methodological choices which reflect different approaches to understanding social influences. For instance, Grotle et al. [53] focus on variables, such as employment status and job dissatisfaction, and use quantitative data from standardised questionnaires. In contrast, Karran et al. [61] use a mixed method to assess a wider range of social influences on CLBP, including socio-economic status and community resources.

Table 2 lists the various ways social factors were measured, mostly through surveys or standardized questionnaires, such as quality of life scales [51, 59, 62, 63, 65, 69, 71] or a specific framework such as the PROGRESS Framework [60]. Recent advances in measurement have seen the introduction of measures more directly related to patient’s experience, like the Patient-Reported Outcomes Measurement Information System (PROMIS) [41] or subjective scales like the McArthur scale, the perceived stress scale, or perceived social isolation [40, 42, 46, 61, 73]. While less frequently used, qualitative methods such as semi-structured interviews, provide a means for exploring the mediating effects and influences of social factors on CLBP [48, 61].

The studies included in this scoping review did not cover the six social domains evenly, with only four of the six being well represented in studies published since 2010. This might be because it is more difficult to assess relationships and social structures using qualitative indicators than the categorical variables used to measure SD, SO or GHD factors, or the standardised psychometric scales used to measure PS factors. However, social relationships, both family and professional, significantly impact the development of CLBP. They affect coping strategies and provide physical and emotional support, which can alleviate mental health issues linked to CLBP. Our data (Fig. 2) clearly show that these factors are underexplored in the literature. Indeed, a safe environment, that provides private and professional social support appears to be essential for recovery, possibly by facilitating acceptance of the situation and by reinforcing coping resources [46]. A favourable work environment in the form of social support from colleagues and the hierarchy might also modulate CLBP outcomes [44, 52]. This is consistent with the Harvard Study of Adult Development, ongoing since 1938, which found that positive social relationships are a fundamental factor in healthier ageing. People who have close and fulfilling relationships with their family and community are generally happier and healthier than those who are socially isolated [89, 90].

### A complementary perspective to existing recommendations

By comparing our results with existing standardized measurement recommendations for CLBP, we observe that certain social factors, such as psychosocial aspects, employment status, and quality of life, are integrated into clinical assessment practices, whereas other dimensions remain underrepresented. The results of an expert consensus process [91, 92] revealed that socio-economic factors are primarily analysed in terms of the financial burden of CLBP (medical expenses, productivity loss) rather than as elements influencing its progression. Similarly, healthcare utilization is assessed mainly in terms of economic impact, without examining its interaction with CLBP. Moreover, these social factors are treated as adjustment variables rather than explanatory ones, thereby limiting their role to that of correcting statistical biases. This approach is also observed in several articles included in our scoping review, where certain factors from the SD and GHD domains are analysed as adjustment variables rather than independent variables [40, 41, 51, 53, 59, 65, 72, 73].

In the recommendations from the Initiative on Methods, Measurement, and Pain Assessment in Clinical Trials (IMMPACT) [93], social factors are not directly assessed. One exception to this is treatment satisfaction, which likely reflects certain aspects of the patient’s experience in relation to the healthcare system. Finally, a recent scoping review [94] broadens the perspective of social factors, by incorporating self-reliance (considered in terms of capacity and performance) and social participation within the framework of the International Classification of Functioning (ICF), although the ICF’s definition lacks conceptual precision, as it does not account for the subjective experience of social participation [95]. Additionally, certain social factors identified in our study, such as coping strategies and employment, were excluded from the Bastiaens et al. assessment, as they were deemed less relevant for the studied population, which is more restricted than ours.

Our findings provide a complementary perspective to current recommendations by highlighting social factors that could be considered in standardized evaluation tools. Our categorisation may thus encourage future research to better characterize these factors and assess their relevance for the development of new clinical recommendations and measurement tools that more comprehensively integrate social dimensions.

### Limitations

Several methodological challenges emerged while performing this scoping review notably related to the potential limitations of our search strategies in capturing relevant literature from social sciences databases. Since these disciplines have different publication systems, like books or proceedings, it can be challenging to find information through electronic databases searching, despite examining databases specific to these fields.

Another difficulty we encountered is that most sociological analyses may not typically operate at the level of CLBP, but rather explore social factors that influence chronic pain. Since we only included studies related to CLBP, we might have overlooked relevant contributions from the broader social sciences literature that could provide valuable insights into the social dimensions of CLBP.

The decision to restrict our review to publications between 2010 and 2023 was based both on practical constraints and on the recent shift in CLBP research toward psychological and social dimensions. While it may have led to us overlooking some relevant literature, investigation of social factors within the BPS model has gained prominence only more recently. As a result, earlier studies are likely limited in number and may not reflect contemporary perspectives shaped by broader societal changes. While this temporal restriction might have affected the comprehensiveness of this scoping review, its impact was likely limited by our deliberately broad and inclusive search strategy.

Furthermore, as recently highlighted by Young [96], it is important to recognise that marginalised populations within Western societies, such as ethnic minorities, individuals from lower socioeconomic classes, and those whose gender identities or sexual orientations fall outside normative frameworks, are often under-represented in health research samples. As a result, the available literature may not adequately capture the influence of marginalisation and the systemic or cultural injustices these populations experience, potentially underestimating their contribution to CLBP. Consequently, the social factors identified in our scoping review likely capture only a portion of a broader and more complex reality.

Finally, we did not conduct a critical appraisal or risk of bias assessment, as this would not align with the objectives of this scoping review. Consequently, we cannot comment on their methodological quality or on the strength of the associations between social factors and the persistence of CLBP.

### Perspectives

A clinical implication of this scoping review is its potential to guide patient care by enhancing healthcare professionals’ awareness of the diverse factors associated with CLBP. This involves distinguishing between individual-level factors and those stemming from institutional or systemic contexts, which may call for broader public health interventions. Tables 3 and 4 can support and structure clinical reasoning by helping guide patients towards the most appropriate care strategies and thereby maximizing their chances of recovery. Additionally, Table 2 provides a list of assessment tools available to healthcare professionals.

Our experience conducting this review has highlighted the need to adopt methodologies that are better aligned with the epistemological and methodological approaches of the humanities and social sciences when studying the social factors of chronic pain. The results of this review highlight a tendency in the literature to frame individuals in terms of categories rather than relationships. A promising avenue for exploring underrepresented domains is the use of mixed methodologies within the participatory action research framework, where participants are engaged in all stages of study design and implementation [97]. This approach would diversify viewpoints and capture the subjective experience of pain, oftentimes overlooked in favour of objective criteria and research outcome indicators. Such an approach might reveal more relevant (but less measurable) factors, particularly interpersonal relationships, and support the development of new tools to assess social influences in the development of CLBP. We observed that social relationships, both personal and professional, interact with various CLBP outcomes, including psychosocial outcomes.

Our review of the literature highlights a clear need for the development of tools to assess key social factors such as interpersonal relationships and social support, and to test their predictive relevance through hypothesis-driven research. A natural extension of this work would be a systematic review with meta-analysis, enabling a more rigorous examination of the temporal and potentially causal links between social factors and the evolution of CLBP.

Another direction for future research is to include the marginalised populations described in the Limitations section, as well as other populations beyond Western, Educated, Industrialized, Rich, and Democratic (WEIRD) societies [98]. Broadening the scope in this way could improve the generalizability of our findings and offer a more comprehensive understanding of CLBP across different sociocultural contexts. For instance, in collectivist cultures—characterized by communal coping mechanisms and strong social networks [99], social support may exert a more pronounced influence on chronic pain management, as compared to individualist societies, where autonomy and self-reliance are culturally emphasized.

## Conclusions

This review provides a comprehensive overview of social factors influencing CLBP, structured into six domains. Our proposed classification provides a foundation for future research and offers clinicians a structured framework to identify relevant factors, at both individual and structural levels. By integrating these elements into care strategies, it may be possible to address modifiable social factors, enhance patients’ capacity for action in managing their condition, and promote more personalized interventions that are better suited to their social and life contexts.

## Supplementary Information

Supplementary file 1

Supplementary file 2

Supplementary file 3

Supplementary file 4

Supplementary file 5

## Data Availability

The datasets generated and/or analysed during the current study are available in the Open Science Framework repository, https://osf.io/z3qkp/?view_only=28b53a46a6d542e7b5f8d3d1aae205f3.

## Abbreviations

CLBP: Chronic Low Back Pain
BPS: Biopsychosocial
JBI: Johanna Briggs Institute
PCC: Population, Concept, Context
SD: Socio-demographic
SO: Socio-occupational
SE: Socio-environmental
GHD: Global Health Data
PS: Psycho-social
PEHC: Patient’s experience of health care
