# Supplementary material for: Social factors contributing to the development of chronic low back pain: a scoping review
Source: BMC Musculoskelet Disord. 2025 Oct 27;26:999. doi: 10.1186/s12891-025-09216-4 (PMC12557928; doi:10.1186/s12891-025-09216-4)
Supplement: Supplementary file 1 [file 12891_2025_9216_MOESM1_ESM.docx]

**Additional file 1**

**Search strategy**

**A complete search strategy is described for Medline**:

#1 Chronic low back pain population :

((("chronic pain*"[Title/Abstract]) OR ("chronic pain*"[MeSH Terms])) OR (("persistent pain"[Title/Abstract])) AND (("low?back pain"[Title/Abstract]) OR ("low?back pain"[MeSH Terms]) OR ("low?back ache*"[Title/Abstract])))

#2 Concepts: biopsychosocial model, psychosocial factors:

("biopsychosocial model*"[Title/Abstract]) OR (Models, Biopsychosocial[MeSH Terms]) OR (bio?psycho?social[Title/Abstract]) OR ("psycho?social factor*"[Title/Abstract]) OR (« psycho?social aspect*"[Title/Abstract]) OR ("situation* factor*"[Title/Abstract]) OR ("social determinant*"[Title/Abstract]) OR (“behavior* factor*”[Title/Abstract])

#3 context:  predictiv assessment :

(prognost*[Title/Abstract]) OR ("predict* factor*"[Title/Abstract]))

The complet search equation:

#1 AND (#2 OR #3):

((("chronic pain*"[Title/Abstract]) OR ("chronic pain*"[MeSH Terms])) OR (("persistent pain"[Title/Abstract])) AND (("low?back pain"[Title/Abstract]) OR ("low?back pain"[MeSH Terms]) OR ("low?back ache*"[Title/Abstract]))) AND (("biopsychosocial model*"[Title/Abstract]) OR (Models, Biopsychosocial[MeSH Terms]) OR (bio?psycho?social[Title/Abstract]) OR ("psycho?social factor*"[Title/Abstract]) OR (« psycho?social aspect*"[Title/Abstract]) OR ("situation* factor*"[Title/Abstract]) OR ("social determinant*"[Title/Abstract]) OR (« behavior* factor* »[Title/Abstract]) OR (prognost*[Title/Abstract]) OR ("predict* factor*"[Title/Abstract]))

**A complete search strategy is described for Cochrane:**

ID Search Hits

#1 MeSH descriptor: [Chronic Pain] explode all trees 4358

#2 MeSH descriptor: [Low Back Pain] explode all trees 5998

#3 (persistent NEAR pain*):ti,ab,kw OR ("chronic low back pain"):ti,ab,kw OR ("recurrent low back pain"):ti,ab,kw OR ("persistent low back pain"):ti,ab,kw OR (chronic NEAR pain*):ti,ab,kw 26261

#4 ("low back pain"):ti,ab,kw OR (low NEAR back NEAR ache*):ti,ab,kw 13651

#5 #1 OR #2 OR #3 OR #4 33122

#6 MeSH descriptor: [Models, Biopsychosocial] explode all trees 2

#7 MeSH descriptor: [Sociological Factors] explode all trees 24364

#8 MeSH descriptor: [Psychology] explode all trees 1487

#9 (biopsychosoci*):ti,ab,kw OR (bio-psycho-soci*):ti,ab,kw OR ("biopsychosocial model"):ti,ab,kw OR (sociological NEAR determin*):ti,ab,kw OR (sociological NEAR facto*):ti,ab,kw 925

#10 (Social NEAR facto*):ti,ab,kw OR (Situational NEAR facto*):ti,ab,kw OR (Social NEAR determin*):ti,ab,kw OR (psychosoci*):ti,ab,kw OR (psychosocial NEAR facto*):ti,ab,kw 24862

#11 #6 OR #7 OR #8 OR #9 OR #10 48295

#12 MeSH descriptor: [Prognosis] explode all trees 204433

#13 ("predictive value"):ti,ab,kw OR (predict*):ti,ab,kw OR (prognos*):ti,ab,kw OR (predictive NEAR facto*):ti,ab,kw OR (Prognostic NEAR facto*):ti,ab,kw 159207

#14 #12 OR #13 324729

#15 #5 ADD #11 ADD #14 4

**A complete search strategy is described for JSTOR:**

(("chronic pain" OR "persistent pain") AND ("low back" OR "low-back")) AND (Biopsychosocial OR bio-psycho-social OR psychosocial OR psycho-social OR social OR situational OR sociologic* OR predict* OR prognos* OR evolut*)

**A complete search strategy is described for Science Direct:**

((("chronic pain"[Title/Abstract]) OR ("persistent pain"[Title/Abstract])) AND (("low?back pain"[Title/Abstract])))

AND

(("bio?psycho?social"[Title/Abstract]) OR ("psycho?social"[Title/Abstract]) OR ("social determinant"[Title/Abstract]) OR (prognos?[Title/Abstract]) OR ("predict? factor?"[Title/Abstract]))

**A complete search strategy is described for Psycinfo:**

**#1**:((**MeSH**: chronic pain *OR* **MeSH**: chronic pains *OR* **MeSH**: pain, chronic *OR* **MeSH**: widespead chronic pain *OR* **MeSH**: pains, chronic *OR* **Title**: "chronic pain*" *OR* **Title**: "persistent pain*" *OR* **Title**: "recurrent pain*" *OR* **Title**: chronic *OR* **Abstract**: "chronic pain*" *OR* **Abstract**: "persistent pain*" *OR* **Abstract**: "recurrent pain*" *OR* **Abstract**: chronic) AND (**MeSH**: low back pain *OR* **MeSH**: low back ache *OR* **MeSH**: ache, low back *OR* **MeSH**: pain, low back *OR* **MeSH**: lower back pain *OR* **Title**: "low back pain" *OR* **Title**: "lower back pain" *OR* **Title**: "low back" *OR* **Title**: "lower back" *OR* **Title**: "back pain" *OR* **Title**: "low back ache*" *OR* **Title**: "lower back ache*" *OR* **Abstract**: "low back pain" *OR* **Abstract**: "lower back pain" *OR* **Abstract**: "low back" *OR* **Abstract**: "lower back" *OR* **Abstract**: "back pain" *OR* **Abstract**: "low back ache*" *OR* **Abstract**: "lower back ache*"))

AND

**#2**:(**MeSH**: Models, Biopsychosocial *OR* **MeSH**: Biopsychosocial model *OR* **MeSH**: biopsychosocial models *OR* **MeSH**: Model, biopsychosocial *OR* **MeSH**: Sociological factors *OR* **MeSH**: Sociological Phenomena *OR* **MeSH**: Sociological factor *OR* **MeSH**: Factor, sociological *OR* **MeSH**: Factors, sociological *OR* **MeSH**: Phenomena, sociological *OR* **Title**: "biopsychosoc*" *OR* **Title**: "bio-psycho-soc*" *OR* **Title**: "biopsychosocial model*" *OR* **Title**: "bio-psycho-social model*" *OR* **Title**: "biopsychosocial approach" *OR* **Title**: "bio-psycho-social approach" *OR* **Title**: psychosoc* *OR* **Title**: "psycho-soc*" *OR* **Title**: "social factor*" *OR* **Title**: "sociological factor*" *OR* **Title**: "social determin*" *OR* **Title**: "sociological determin*" *OR* **Title**: "situational factor*" *OR* **Title**: "biopsychosocial factor*" *OR* **Title**: "psychosocial factor*" *OR* **Title**: "bio-psycho-social factor*" *OR* **Title**: "psycho-social factor*" *OR* **Title**: "sociological phenomena" *OR* **Title**: "social threat" *OR* **Abstract**: "biopsychosoc*" *OR* **Abstract**: "bio-psycho-soc*" *OR* **Abstract**: "biopsychosocial model*" *OR* **Abstract**: "bio-psycho-social model*" *OR* **Abstract**: "biopsychosocial approach" *OR* **Abstract**: "bio-psycho-social approach" *OR* **Abstract**: psychosoc* *OR* **Abstract**: "psycho-soc*" *OR* **Abstract**: "social factor*" *OR* **Abstract**: "sociological factor*" *OR* **Abstract**: "social determin*" *OR* **Abstract**: "sociological determin*" *OR* **Abstract**: "situational factor*" *OR* **Abstract**: "biopsychosocial factor*" *OR* **Abstract**: "psychosocial factor*" *OR* **Abstract**: "bio-psycho-social factor*" *OR* **Abstract**: "psycho-social factor*" *OR* **Abstract**: "sociological phenomena" *OR* **Abstract**: "social threat")

AND

**#3**:(**MeSH**: prognosis *OR* **MeSH**: prognoses *OR* **MeSH**: prognostic factors *OR* **MeSH**: factor, prognostic *OR* **MeSH**: factors, prognostic *OR* **MeSH**: prognostic factor *OR* **Title**: "predictive value" *OR* **Title**: predict* *OR* **Title**: prognos* *OR* **Title**: "prognostic factor*" *OR* **Title**: "predictive factor*" *OR* **Title**: "prognosis factor*" *OR* **Abstract**: "predictive value" *OR* **Abstract**: predict* *OR* **Abstract**: prognos* *OR* **Abstract**: "prognostic factor*" *OR* **Abstract**: "predictive factor*" *OR* **Abstract**: "prognosis factor*" )

**A complete search strategy is described for Web of Science:**

**#1** : TS=("low back pain" OR "recurrent low back pain" OR "persistent low back pain" OR "chronic low back pain" OR "low back ache*" OR "chronic low back ache*") AND TS=("chronic pain*" OR "persistent pain*" OR "widespread chronic pain" )

**#2**: TS=(biopsychosocial OR bio-psycho-social OR "biopsychosocial model*" OR "bio-psycho-social model*" OR "biopsychosocial facto*" OR "bio-psycho-social facto*" OR psychosocial OR psycho-social OR "psychosocial facto*" OR "psycho-social facto*" OR "social facto*" OR "social determin*" OR "sociological facto*" OR "sociological determin*" OR "situational facto*" OR "social threa*" )

**#3**: TS=("predictive value" OR predict* OR prognos* OR "prognostic facto*" OR "predictive facto*" OR "prognosis facto*")

#1 AND #2 AND #3

<https://www.webofscience.com/wos/woscc/summary/602bd7fd-fe69-41dd-b591-a2d02326ee08-cb6cf6dd/relevance/1>

**A complete search strategy is described for CAIRN :**

(("low back pain" OR "recurrent low back pain" OR "persistent low back pain" OR "chronic low back pain" OR "low back ache*" OR "chronic low back ache*") AND ("chronic pain*" OR "persistent pain*" OR "widespread chronic pain")

AND

(biopsychosocial OR bio-psycho-social OR "biopsychosocial model*" OR "bio-psycho-social model*" OR "biopsychosocial facto*" OR "bio-psycho-social facto*" OR psychosocial OR psycho-social OR "psychosocial facto*" OR "psycho-social facto*" OR "social facto*" OR "social determin*" OR "sociological facto*" OR "sociological determin*" OR "situational facto*" OR "social threa*" )

AND

("predictive value" OR predict* OR prognos* OR "prognostic facto*" OR "predictive facto*" OR "prognosis facto*"))

OR

(("lombalgie chronique" OR "lombalgie persistante" OR "lombalgie non-spécifique chronique" OR "douleurs de dos chroniques" OR "douleurs de dos persistantes")

AND

("modèle biopsychosocial" OR "modèle bio-psycho-social" OR biopsychosocial OR bio-psycho-social OR "facteurs psychosociaux" OR "facteurs sociologiques" OR "facteurs sociaux" OR "déterminants sociaux")

AND

("prédictif" OR "pronostic" OR "facteur prédictif" OR "facteur pronostic"))

**A complete search strategy is described for Google Scholar:**

((("chronic pain*" OR "persistent pain*") AND ("low back pain" OR "low back ache"))

AND

(Biopsychosocial OR bio-psycho-social OR "biopsychosocial model*" OR "bio-psycho-social model*" OR Psychosocial OR Psycho-social OR "Social facto*" OR "Social determin*" OR "Situational facto*" OR "Social stimu*" OR "Biopsychosocial facto*" OR "Psychosocial facto*" OR "Psycho-social facto*" OR "Sociological determin*" OR "Sociological Facto*" OR "Bio-psycho-social facto*" OR "Sociological phenom*" OR "Bio-psycho-social appr*" OR "Biopsychosocial appr*" OR "Social threa*" OR "Predictive Value" OR Predict* OR Prognos* OR "Prognostic factor*" OR "Predictive factor*" OR "Prognosis factor*"))

OR

((("douleur chronique" OR "douleur persistante" OR "douleurs chroniques" OR "douleurs persistantes") AND ("lombalgie chronique" OR "douleurs de dos persistante*" OR "lombalgie chronique"))

AND

(Biopsychosocial OR bio-psycho-social OR Psychosocial OR Psycho-social OR "facteurs soci*" OR "déterminants soci*" OR "Situational facto*" OR "Social stimu*" OR "Biopsychosocial facto*" OR "Psychosocial facto*" OR "Psycho-social facto*" OR "Sociological determin*" OR "Sociological Facto*" OR "Bio-psycho-social facto*" OR "déterminants sociaux de la santé" OR "facteurs situationnels" OR "Biopsychosocial appr*" OR "menace sociale" OR "valeur prédictive" OR Predict* OR Pronos* OR "facteur pronosti*" OR "facteur predictif*" OR "facteurs pronosti*"))
