# Supplementary material for: Social factors contributing to the development of chronic low back pain: a scoping review
Source: BMC Musculoskelet Disord. 2025 Oct 27;26:999. doi: 10.1186/s12891-025-09216-4 (PMC12557928; doi:10.1186/s12891-025-09216-4)
Supplement: Supplementary file 2 [file 12891_2025_9216_MOESM2_ESM.docx]

**Additional file 2**

**Data extraction instrument**

This data charting instrument is based on the JBI guidelines. It was adapted to the needs of the current scoping review to provide the most relevant data selection.

| **AUTHORS** | Name of first authors, if more than three authors, use of “et al.” |
| --- | --- |
| **TITLE** | Title of the article |
| **YEAR OF PUBLICATION** | The year the article was published |
| **NAME OF PUBLICATION / JOURNAL** | Where was the article published |
| **PRIMARY AFFILIATION FIRST AUTHOR** | The affiliation of the first author |
| **TYPE OF EVIDENCE SOURCE** | E.g. Primary research, Epidemiology, Literature review, Guidelines... |
| **AIM OF ARTICLE** | Main objective of the article |
| **METHODOLOGY*** | E.g. Quantitative, Qualitative, Mixed-methods |
| **STUDY DESIGN*** | E.g. Clinical Trial, Observational Study, prospective, retrospective, phenomenology, ethnography... |
| **DATA COLLECTION*** | E.g. Clinical records, Surveys, Interviews... |
| **DATE THE DATA COLLECTION*** | Time period of data collection |
| **LOCATION STUDY DATA WAS COLLECTED** | Country where the data were collected |
| **POPULATION** | Description of population data |
| **CONCEPT** | Description of the concept of article |
| **CONTEXT** | Description of the context of article |
| **SAMPLE SIZE*** | Participant sample size |
| **INTERVENTION TYPE*** | The intervention used in the trial |
| **COMPARATOR*** | The control method used for the intervention |
| **PRIMARY OUTCOME*** | How the main objective was measured |
| **SECONDARY OUTCOMES*** | How the secondaries objectives were measured |
| **KEYFINDINGS THAT RELATE TO THE QUESTION** | Qualitative data that relate to the question |
| **COMMENTS** | If necessary, reviewer comments |

^*NA if not relevant or not applicable^
