# Supplementary material for: Social factors contributing to the development of chronic low back pain: a scoping review
Source: BMC Musculoskelet Disord. 2025 Oct 27;26:999. doi: 10.1186/s12891-025-09216-4 (PMC12557928; doi:10.1186/s12891-025-09216-4)
Supplement: Supplementary file 3 [file 12891_2025_9216_MOESM3_ESM.docx]

**Additional file 3**

**Citation network**

method used to evaluate the citation network and create the map based on Zotero7

- <https://forums.zotero.org/discussion/78671/citation-mapping-network-map-of-zotero-library>
- <https://github.com/jaks6/citation_map>
