# Supplementary material for: Social factors contributing to the development of chronic low back pain: a scoping review
Source: BMC Musculoskelet Disord. 2025 Oct 27;26:999. doi: 10.1186/s12891-025-09216-4 (PMC12557928; doi:10.1186/s12891-025-09216-4)
Supplement: Supplementary file 4 [file 12891_2025_9216_MOESM4_ESM.docx]

To find the selection of articles table, follow this link to the Open Science Framework page of the scoping review:

<https://osf.io/z3qkp/?view_only=28b53a46a6d542e7b5f8d3d1aae205f3>
